# The clinical significance of long non-coding RNAs MALAT1 and CASC2 in the diagnosis of HCV-related hepatocellular carcinoma

**DOI:** 10.1371/journal.pone.0303314

**Published:** 2024-05-13

**Authors:** Rehab M. Golam, Mahmoud A. F. Khalil, Olfat G. Shaker, Tarek I. Ahmed, Mohamed K. Abd Elguaad, Essam A. Hassan, Mahmoud R. M. El-Ansary, Ahmed Ismail, Yasser I. Kandil, Osama A. Mohammed, Ahmed S. Doghish

**Affiliations:** 1 Department of Medical Biochemistry and Molecular Biology, Faculty of Medicine, Fayoum University, Fayoum, Egypt; 2 Department of Microbiology and Immunology, Faculty of Pharmacy, Fayoum University, Fayoum, Egypt; 3 Department of Medical Biochemistry and Molecular Biology, Faculty of Medicine, Cairo University, Cairo, Egypt; 4 Department of Internal Medicine, Faculty of Medicine, Fayoum University, Fayoum, Egypt; 5 Department of Medical Physiology, Faculty of Medicine, Fayoum University, Fayoum, Egypt; 6 Department of Tropical Medicine, Faculty of Medicine, Fayoum University, Fayoum, Egypt; 7 Department of Medical Microbiology and Immunology, Faculty of Medicine, Misr University for Science and Technology (MUST), Giza, Egypt; 8 Biochemistry and Molecular Biology Department, Faculty of Pharmacy (Boys), Al-Azhar University, Nasr City, Cairo, Egypt; 9 Department of Biochemistry, Faculty of Pharmacy, Sinai University–Kantara Branch, Ismailia, Egypt; 10 Department of Pharmacology, College of Medicine, University of Bisha, Bisha, Saudi Arabia; 11 Department of Biochemistry, Faculty of Pharmacy, Badr University in Cairo (BUC), Badr City, Cairo, Egypt; 12 Faculty of Pharmacy (Boys), Al-Azhar University, Nasr City, Cairo, Egypt; iBiMED - Institute of Biomedicine, PORTUGAL

## Abstract

**Background:**

Globally, hepatocellular carcinoma (HCC) is the second most common cause of cancer-related death due to a lack of early predictive and/or diagnostic tools. Thus, research for a new biomarker is important. LncRNAs play a functional role in target gene regulation and their deregulation is associated with several pathological conditions including HCC.

**Objective:**

This study aimed to explore the diagnostic potential of two LncRNAs MALAT1 and CASC2 in HCC compared to the routinely used diagnostic biomarker.

**Materials and methods:**

The current study is a case-control study carried out at Fayoum University Hospital and conducted on 89 individuals. The study included three groups of 36 HCC patients on top of HCV(HCC/HCV), 33 HCV patients, and 20 healthy volunteers as a control group. All study subjects were subjected to radiological examinations. The determination of CBC was performed by the automated counter and liver function tests by the enzymatic method were performed. In addition, HCV RNA quantification and the expression level of two LncRNAs (MALAT1 and CASC2) were performed by qRT-PCR.

**Results:**

The results revealed a statistically significant difference between study groups regarding liver function tests with a higher mean in HCC/HCV group. Also, serum MALAT1 significantly up-regulated in HCV (11.2±2.8) and HCC/HCV (4.56±1.4) compared to the control group. Besides, serum CASC2 levels in the HCV group were significantly upregulated (14.9±3.6), while, downregulated in the HCC group (0.16± 0.03). Furthermore, The ROC analysis for diagnostic efficacy parameters indicated that CASC2 has higher accuracy (94.6%) and sensitivity (97.2%) for HCC diagnosis than AFP with an accuracy of (90.9%), sensitivity (69.4%), and MALAT1 showed an accuracy of (56.9%), sensitivity (72.2%).

**Conclusion:**

Our study results indicated that CASC2 is a promising biomarker and is considered better and could help in HCC diagnosis on top of HCV than MALAT1 and the routine biomarker AFP.

## Introduction

Hepatocellular carcinoma (HCC) is the world’s fifth most prevalent malignancy and the second most common cause of cancer-related death [1]. Finding new biomarkers for disease diagnosis or predicting the clinical outcome is of vital importance [2, 3]. Infection with the HCV virus is one of the most common causes of HCC [4]. LncRNAs (long non-coding RNAs) are RNAs with a length of more than 200 nucleotides, initially, they were discovered as mRNA-like transcripts without protein-coding capacity. LncRNAs are arranged in secondary and three-dimensional structures so they can function as RNA and or protein [5]. The identification of non-coding RNA, such as long non-coding RNA (lncRNA) and microRNA (miRNA), in the advancement of human diseases has broadened to encompass potential connections with various conditions [6–10].

**Fig 1:**
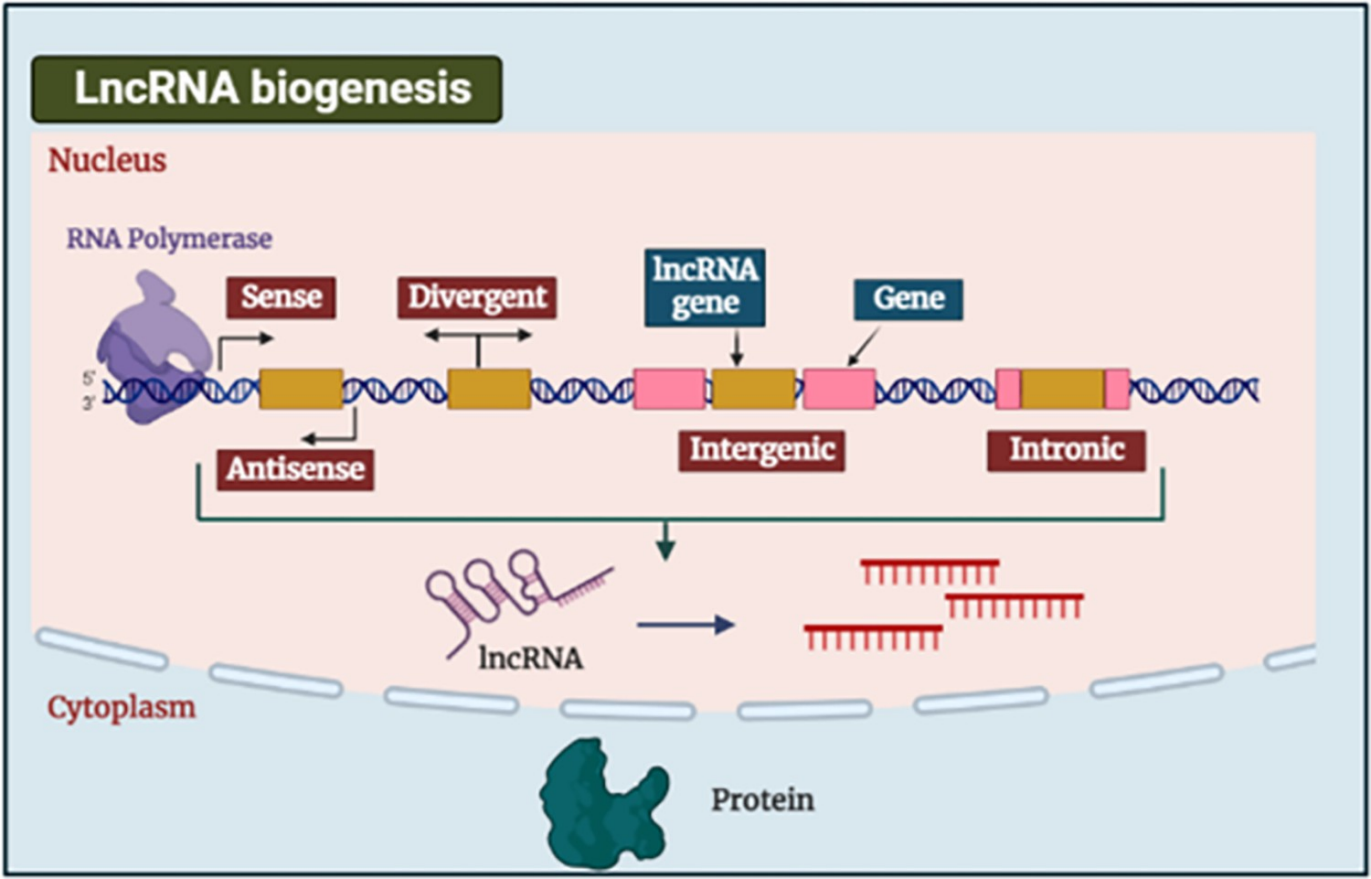
Biogenic pathways of lncRNAs. LncRNAs can behave like an oncogene-long noncoding RNA (OncoLncs) [14] or as a tumor suppressor long noncoding RNA (TSLncs) [15, 16]. Non-coding RNAs, such as lncRNAs and miRNAs, are now known to have a role in a wide variety of processes that lead to a wide variety of disorders [17]. LncRNAs serve as potential diagnostic biomarkers for HCC with high sensitivity and specificity [18]. Metastasis-associated lung adenocarcinoma transcript 1 (MALAT1) and cancer susceptibility candidate 2 (CASC2) are LncRNA that are dysregulated in HCC tissue [19, 20].

LncRNAs participate in the regulation of target gene expression, through cis or trans-regulation [11]. They play an essential role in many physiological processes and their dysregulation is associated with many pathological conditions like cancer [12]. lncRNAs are classified into several categories based on their chromosomal location, including sense, anti-sense, bidirectional, intronic, and intergenic. They undergo splicing, 5′ capping, and polyadenylation after transcription by RNA polymerases. While some lncRNAs are translated into proteins, the vast majority play roles in genome control or as binding partners for molecules either inside (e.g., transcripts like microRNAs (miRNAs)) or outside (e.g., proteins) the nucleus immediately after transcription [13] (**Fig 1**).

LncRNA MALAT1 also known as NEAT-2 (nuclear enriched abundant transcript-2) or LINC00047, was initially characterized by Ji and coworkers (2003) in non-small cell lung cancer [21], it acts as a proto-oncogene and is located at 11q13 [22]. CASC2 is long noncoding RNA identified in chromosome 10 open reading frame 5, it acts as a tumor suppressor. It was initially described by Baldinu and co-workers in 2004 in the endometrial carcinoma cell [23]. Down-regulation of CASC2 in HCC tissue and cell lines was approved in many previous reports [20, 24].

The authors have thoughtfully selected the lncRNAs MALAT1 and CASC2 for further study based on several key criteria. Firstly, these lncRNAs exhibit conserved expression patterns, suggesting they may play important regulatory roles. Additionally, the authors note that lncRNAs tend to have highly cell-type-specific expression, which can complicate the detection of associated phenotypes [25]. By focusing on lncRNAs near protein-coding genes of interest, the researchers aim to increase the chances of observing meaningful functional effects. The authors also acknowledge the potential for functional redundancy among lncRNAs, highlighting the importance of careful experimental design. Overall, the selective approach to studying MALAT1, CASC2, and other promising lncRNA candidates reflects the authors’ efforts to overcome the challenges inherent in this rapidly expanding field of research, to uncover the biological functions and mechanisms of action for this class of transcripts.

This study aims to evaluate the diagnostic efficacies of the oncogenic long noncoding RNA MALAT1 and the tumor suppressor CASC2 as potential biomarkers for HCC on top of HCV in comparison to routinely used biomarkers.

## Patients and methods

### Study design

The current study is a case-control study, conducted on 89 individuals of both genders. The study included three groups of 36 HCC patients on top of HCV(HCC/HCV), 33 HCV patients, and 20 healthy volunteers as a control group. The control group was selected from healthy blood bank donors. They have normal liver enzyme range, normal liver ultrasound examination, absence of congenital or infectious disease affecting the liver, and no history of cancer affection. All patients were recruited from Fayoum University Hospital in the period from March 3, 2023, to September 12, 2023. The studied groups had matched ages and gender.

To confirm a diagnosis of HCV and exclude HBV infection, the key steps were as follows. First, serological testing was performed to screen for HCV antibodies (anti-HCV). A positive anti-HCV result indicates prior exposure to HCV but does not confirm active infection. To confirm active HCV infection, a nucleic acid test was then conducted to detect the presence of HCV RNA. A positive HCV RNA test confirms chronic HCV infection and was necessary before considering treatment. To rule out active HBV infection, the patient was also screened for the hepatitis B surface antigen (HBsAg).

### Ethical approval

Based on the ethical guidelines of the Declaration of Helsinki, the study protocol was reviewed and approved by the Research Ethics Committee of the Faculty of Medicine, Fayoum University (Permit No. R 407). The study complies with all regulations and written informed consent was obtained from all participants before the withdrawal of the blood sample.

### Blood samples and investigations

Five mL of venous blood samples were collected from patients and the control group using the BD Vacutainer system into two serum-separating tubes and one EDTA-containing tube. The serum-separating tubes were allowed to clot and centrifuged at 4000 rpm for 10 min. Obtained sera were divided into aliquots and stored at −80°C. One aliquot was used for the determination of liver function tests, and HCV RNA detection to confirm HCV infection and exclude HBV. The second aliquot was used for AFP determination. The third aliquot was used for LncRNA expression assay by QRT-PCR. The EDTA-containing tube was used for complete blood count determination. The sample size was calculated using G-power software (version 3.1.9.7; Heinrich-Heine-Universität Düsseldorf, Düsseldorf, Germany)

### All groups were subjected to abdominal ultrasound examination and classified into:

**1. Control group**: excluding any liver problems such as hepatomegaly, fibrosis, and hepatic mass.

**2. HCV patients**: assessment of fibrosis and cirrhosis excluding hepatic mass.

**3. HCC/HCV patient:** further CT imaging for assessment of liver mass.

### Laboratory investigations

The serum alanine transaminase (ALT), serum aspartate transaminase (AST), alkaline phosphatase (ALP), serum bilirubin (total and direct bilirubin), and albumin analyses were carried out by an enzymatic method. Alanine aminotransferase (ALT) (Item No. 11533), Aspartate aminotransferase (AST) (Item No. 11531), Alkaline phosphatase (ALP) (Item No.11592), and bilirubin (Item No. 11515) assay kits were purchased from BioSystems S.A., Barcelona (Spain).

Serum AFP concentration was measured by the chemiluminescent immunometric assay on Siemens IMMULITE® 2000 (Siemens Healthcare Diagnostics, USA). An automated cell counter (Cell Dyne-2700, Abbott Lab, USA) was used for hemoglobin (Hb) estimation, white blood cell (WBC) count, and platelet count detection.

### LncRNA expression assay

#### RNA extraction

RNA extraction from serum was performed using miRNeasy extraction kit (Qiagen, Valencia, CA, USA) QIAzol lysis reagent according to the manufacturer’s protocol. To determine the quality of the extracted RNA NanoDrop (ND)-1000 spectrophotometer (NanoDrop Technologies, lnc., Wilmington, DE, USA) was used.

#### Quantitative reverse transcriptase‐polymerase chain reaction (qRT‐PCR) of lncRNA (MALAT1 and CASC2)

RNA was reverse-transcribed using a reverse transcriptase RT2 first strand Kit (Qiagen, Valencia, CA, USA). Quantitative Real-time PCR was done for long noncoding RNA, MALAT1, and CASC2 using GAPDH as internal control all provided by Qiagen. The primer sequences of each are seen in **Table 1**.

**Table 1:** Primer sequences for studied LncRNAs and internal control.

Target	Forward primer sequences	Reverse primer sequences
**LncRNA MALAT1**	5′-AAA GCA AGG TCT CCC CAC AAG-3′	5′-GGT CTG TGC TAG ATC AAA AGG C-3′
**lncRNA CASC2**	5′-GCA CAT TGG ACG GTG TTT CC-3′,	5′-CCC AGT CCT TCA CAG GTC AC-3′
**GAPDH**	5′-GGT GGT CTC CTC TGA CTT CAA CA-3′	5′-GTG GTC GTT GAG GGC AAT G-3′

Maxima SYBR Green PCR kit was used for amplification (Thermo, USA). The PCR mix 20 μl (10 μl Maxima SYBR Green,1 μl forward primer, 1 μl reverse primer, 2.5 μl cDNA, and 5.5 μl RNAase-free water). Cycling conditions consist of holding stages at 95°C for 10 min, followed by 45 cycles at 95°C for 15 s and 60°C for 60 s.

#### Gene expression and fold change determination

Gene expression relative to internal control was calculated using the (2^-ΔCt^) formula. The cycle threshold (Ct) is the number of cycles required for the fluorescent signal to cross the threshold in quantitative real-time PCR. The fold change was calculated using the 2^-ΔΔCt^ formula [26, 27].

### Statistical analysis

All outcomes were examined using GraphPad Prism 8 software (GraphPad, San Diego, USA). Data passed the Shapiro-Wilk normality test and were shown as means and standard deviations (SD). To examine the statistically significant difference between the outcomes of all groups, Tukey post hoc multiple comparison tests were used in conjunction with a one-way analysis of variance (One-way ANOVA). It was deemed statistically significant at P < 0.05.

## Results

### Study groups and patients’ criteria

The Study groups included 36 HCC cases on top of HCV (HCC/HCV), 33 HCV cases, and 20 healthy volunteers. The HCC/HCV group includes 28 males and 8 females, with a mean age of 59.8±1.2. The HCV group included 24 males and 9 females with an age mean of 60.1±1.0. The control group comprised 13 males and 7 females, and the mean age was 59.3±1.6 (**Table 2**).

**Table 2:** Comparisons of routine investigations and, liver function tests in different study groups.

Variables	HCC/HCV (n = 36)		HCV (n = 33)		Control (n = 20)		p-value
**Sex**							
**Males**	28	78%	24	73%	13	65%	0.6
**Females**	8	22%	9	27%	7	35%	
**Age (year)**	Mean±SD		Mean±SD		Mean±SD		
	59.8±1.2		60.1±1.02		59.3±1.6		0.9
**Liver function tests**							
**AST (IU/L)**	117.4±10.5		61.4±7.1		39.2±0.9		<0.001*
**ALT (IU/L)**	76±8		69.3±8.8		44.4±2.1		0.03*
**ALP (IU/L)**	178.2±24.8		100.2±8.9		46.4±2.3		0.006*
**Total bilirubin (mg/dl)**	1.8±0.3		0.75±0.05		0.35±0.04		0.001*
**Direct bilirubin(mg/dl)**	0.8±0.2		0.41±0.2		0.26±0.2		0.002*
**Albumin (g/dl)**	3.8±0.2		4.5±0.2		4.2±0.04		0.04*
**AFP (ng/mL)**	224 ±910		6.3±1.5		2.4±0.2		0.01*
**Complete blood count**							
**HB**	11.3±0.4		13.2±0.2		13.7±0.2		<0.001*
**WBC**	5.8±0.4		6.1±0.5		4.4±0.1		0.04*
**PLT**	126.4±13.3		236.9±11.7		242.8±10.9		<0.001*

*significant difference observed between the three groups

### Biochemical laboratory findings among studied groups

**Table 2** also illustrates the Hb, WBCs, Platelets, liver function tests, and AFP in study groups. We observed a low mean of hemoglobin concentration and platelet count among the HCC group with statically significant differences between the studied groups. A high mean of WBCs was detected among the HCV group with significant differences between groups. There was a statistically significant difference with a p-value <0.05 between the different groups (HCC/HCV, HCV, and control) as regards liver function tests. High means of liver enzymes, bilirubin level, and AFP were reported among the HCC/HCV group, on the other hand, a low mean of albumin level was noted.

### Serum fold change of MALAT1 and CASC2 in study groups

**Fig 2:**
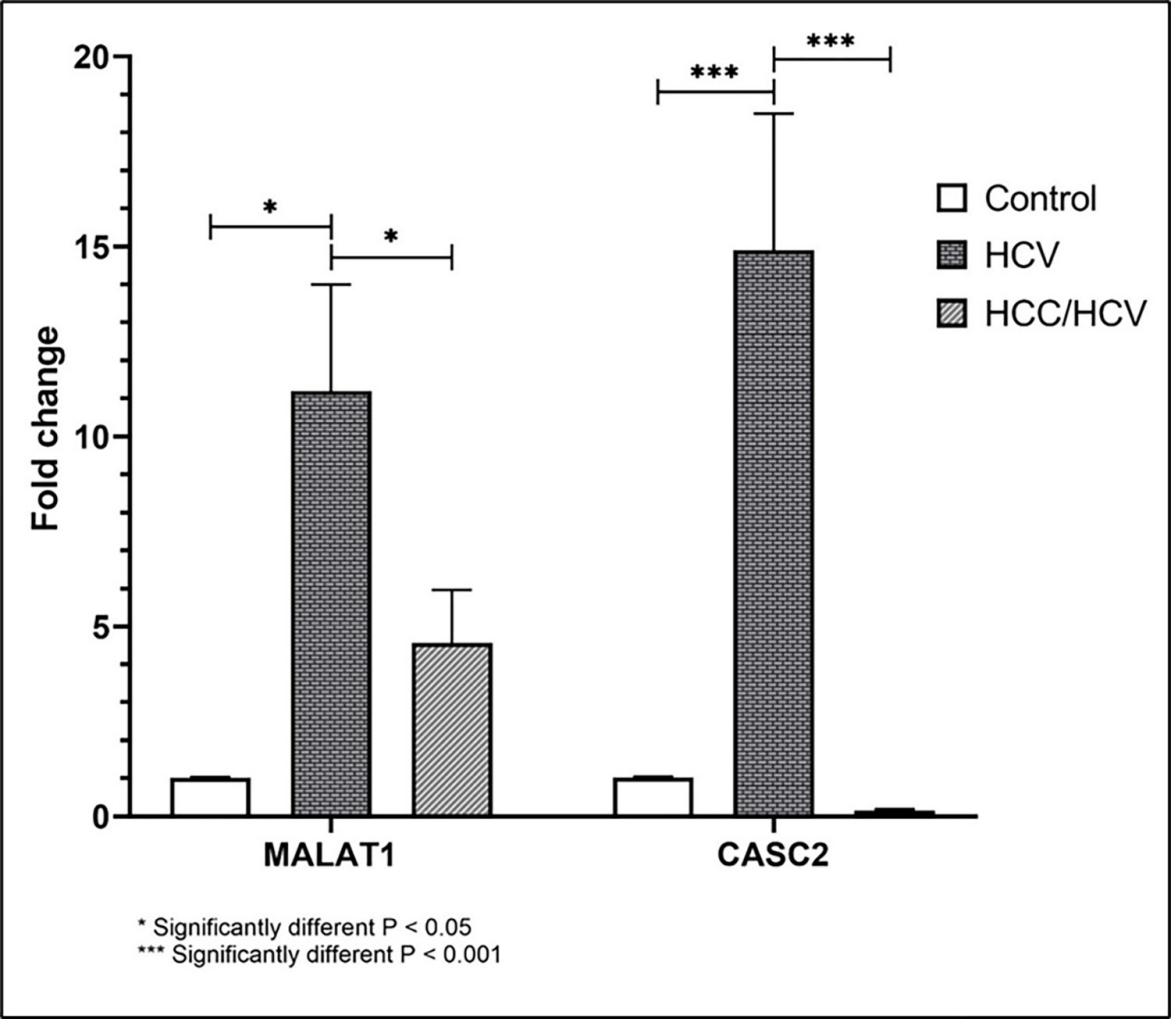
Comparisons of lncRNA MALAT1 and CASC2 fold change in blood of different study groups.

**Table 3** and **Fig 2** illustrate the fold change for MALAT1 and CASC2 in study groups. Serum MALAT1 fold change in HCV group patients showed significant up-regulation (more than 2-fold change) (MALAT1 = 11.2±2.8) compared to the control group. MALAT1 had the highest fold change in the HCV group compared to other groups. Additionally, serum MALAT1 levels in HCC/HCV group patients had numerically higher fold change (MALAT1 = 4.56±1.4) than in the control group but lower than that of the HCV group with a significant difference between the groups. Besides, serum CASC2 levels in HCV group patients showed significant up-regulation in the HCV group (CASC2 = 14.9±3.6) compared to the control. Furthermore, serum CASC2 levels in HCC/HCV group patients showed significant down-regulation (CASC2 = 0.16± 0.03) in comparison to both the HCV group and control. There was a statistically significant difference between the three groups (p = <0.001*).

**Table 3:** Comparisons of lncRNA MALAT1 and CASC2 fold change in blood of different study groups.

Variables	HCC/HCV (n = 36)	HCV (n = 33)	Control (n = 20)	p-value
	Mean±SD	Mean±SD	Mean±SD	
**Biomarkers**				
**MALAT1**	4.56±1.4	11.2±2.8	1.01±0.03	0.008*
**CASC2**	0.16±0.03	14.9±3.6	1.03±0.02	<0.001*

*significant difference observed between the three groups

**MALAT1**: Metastasis-associated lung adenocarcinoma transcript 1; **CASC2**: Cancer susceptibility candidate 2.

### MALAT1 and CASC2 biomarker deregulation in HCV and HCC/HCV groups

Regarding MALAT1, 58% of HCV patients showed up-regulation, while half of HCC patients were up-regulated, while, and the other half were down-regulated **(Table 4)**. Regarding CASC2, 60% of HCV patients showed up-regulation, while nearly all HCC patients showed down-regulation **(Table 4)**.

**Table 4:** LncRNAs MALAT1 and CASC2 markers deregulated expression among HCV and HCC study group.

Variables	MALAT1		CASC2	
	HCC/HCV 36	HCV 33	HCC/HCV 36	HCV 33
	No. (%)	No. (%)	No. (%)	No. (%)
**Similar to control**	3 (8%)	4 (10%)	1 (3%)	0 (0%)
**Up-regulated**	18 (50%)	22 (58%)	0 (0%)	23 (60%)
**Down-regulated**	15 (42%)	12 (32%)	35 (97%)	15 (40%)

**Up-regulation** (2-fold change increase in comparison to control); **Downregulation** (2-fold change decrease in comparison to control decrease of 50% between two measurements).

### Relation between radiological (CT) assessment of HCC/HCV group and MALAT1 and CASC2 serum level of patients

No significant relation was found between MALAT1, and CASC2 serum level in the HCC/HCV group and CT finding (Number or size of liver foci, liver or spleen size, Ascites level, and portal vein state) **(Table 5)**.

**Table 5:** CT finding in the HCC group and its relation to MALAT1 and CASC2 serum level.

Variables	HCC/HCV (n = 36)		MALAT1	p-value	CASC2	p-value
	Number	%	Mean ± SD		Mean ± SD	
**Number of liver foci**						
Single	23	63.9%	3.03±1	0.3	0.15±0.03	0.8
Multiple	13	36.1%	7.3±3.5		0.17±0.07	
**Size of largest foci**						
≤ 5 cm	18	50%	5.9±2.5	0.4	0.18±0.06	0.6
>5cm	18	50%	3.2±1.3		0.14±0.03	
**Liver size**						
Shrunken	3	8.3%	3.2±2.4	0.9	0.21±0.1	0.4
Average	15	41.7%	5.5±2.6		0.21±0.07	
Enlarged	18	50%	4.01±1.9		0.11±0.03	
**Portal vein**						
Patent	27	75%	5.2±1.9	0.5	0.15±0.04	0.7
Thrombosis	9	25%	2.8±1.04		0.18±0.07	
**Spleen size**						
Average	20	55.6%	3.5±1.6	0.4	0.13±0.04	0.4
Enlarged	16	44.4%	5.9±2.6		0.19±0.07	
**Ascites level**						
No	24	66.7%	5.9±2.1	0.6	0.17±0.05	0.9
Mild	5	13.9%	3.4±1.3		0.13±0.01	
Moderate	5	13.9%	1.1±0.3		0.16±0.1	
Sever	2	5.6%	0.57±0.2		0.063±0.04	

### ROC analysis of MALAT1 and CASC2 for HCC diagnosis

**Fig 3:**
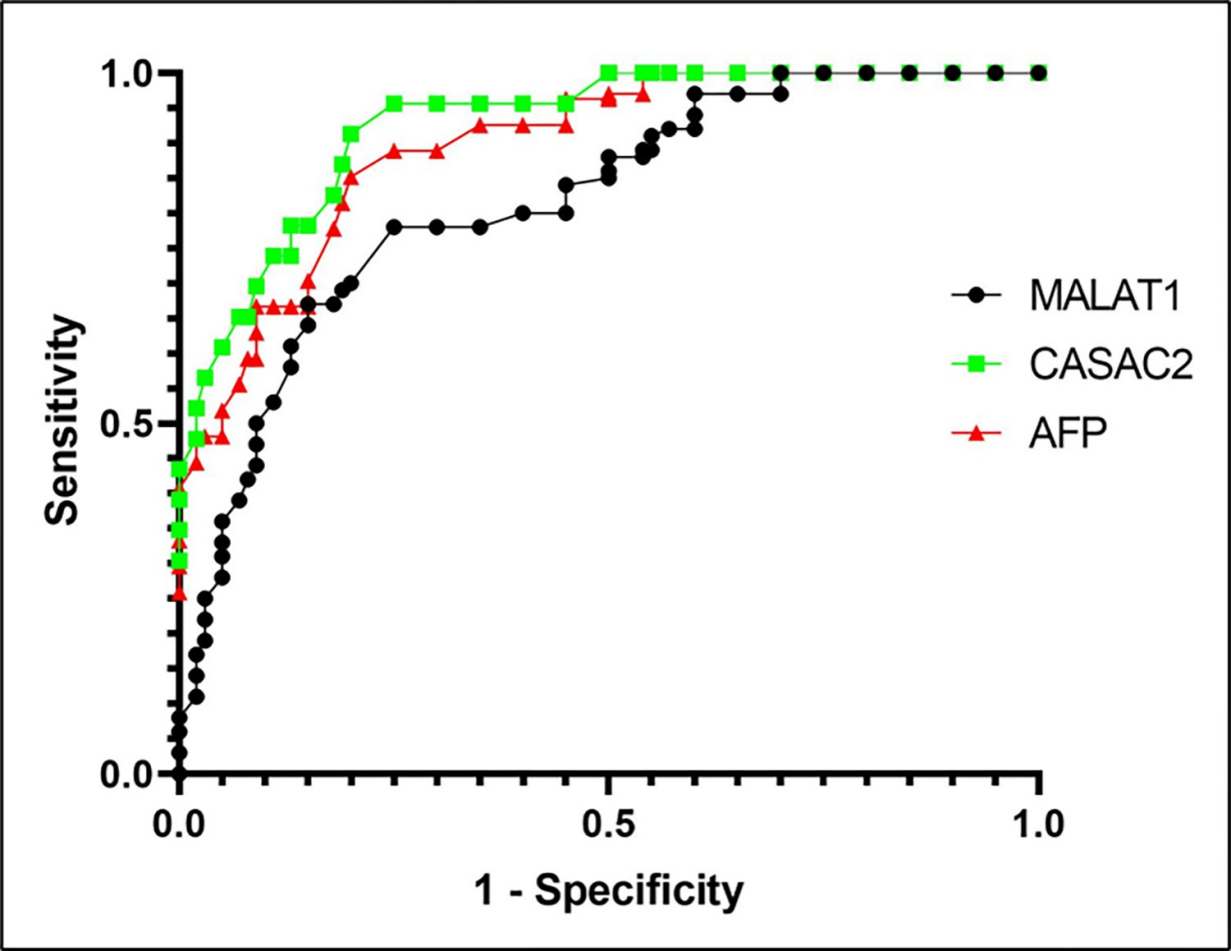
ROC analysis of MALAT1 and CASC2 for HCC diagnosis.

ROC analysis was performed to determine the diagnostic efficacy and the cut‐off value for the two LncRNAs and the routine biomarker AFP. The data revealed that MALAT1 showed an accuracy of 56.9%, a sensitivity of 72.2%, and a specificity of 55.4% at the cutoff level of 2.65. Roc curve of CASC2 showed an accuracy of 94.6% with a sensitivity of 97.2% and specificity of 60.5% at a cutoff level of 0.995. While, the routine biomarker AFP has an accuracy of 90.9%, a sensitivity of 69.4%, and a specificity of 97% at the cutoff level of 28.55. These data indicated that CASC2 has higher accuracy and sensitivity for HCC diagnosis than AFP. On the other hand, AFP has a higher specificity for HCC diagnosis than CASC2 **Table 6 and Fig 3**.

**Table 6:** Sensitivity and specificity of MALAT and CASC2 marker in the diagnosis of HCC.

Variable	Sensitivity	Specificity	Accuracy	Cut off point
**MALAT1**	**72.2%**	**55.4%**	**56.9%**	**2.65**
**CASC2**	**97.2%**	**60.5%**	**94.6%**	**0.995**
**AFP**	**69.4%**	**97%**	**90.9%**	**28.55**

## Discussion

HCC incidence showed a four-fold increase over the last four decades and it may continue to rise in the future [28]. Although different therapeutic options are available for HCC patients, HCC patients showed the absence of symptoms in the early stages so they remained undiagnosed until the late stages [29]. This limits the opportunities for successful intervention [30]. Early and accurate diagnosis through using single or combined tumor markers including specifically expressed proteins, miRNAs, LncRNAs, and epigenetic markers will improve the opportunity for proper treatment. The researcher’s endeavors have to continue to dig in for new markers and evaluate them to find the perfect combination for accurate diagnosis. We evaluate two long noncoding RNAs, CASC2 and MALAT1 as new promising biomarkers for HCC. The marker to be used for HCC diagnosis should consider the pre-pathological state of the liver such as the grade of cirrhosis, which can affect the biomarker level as well as its specificity and sensitivity.

HCC diagnosis is complicated due to the coexistence of inflammation and cirrhosis. It was noticed that the plasma level in HCC patients was affected by their pre-state. Where MALAT1 levels in HCC patients with HBV infection were significantly lower than those with HCV infection [31]. Our study was designed to include healthy volunteers (control group), they represent the baseline for biomarker serum expression. HCV group without HCC was involved in our report to investigate HCC pre-state. HCV is a serious predisposing factor for HCC development [4]. HCV affects 2%–3% of the world’s population [32]. Egypt is plagued with the highest rate of HCV prevalence and the government raised a national campaign in 2019 for detecting and treating HCV patients [33].

Many reports have approved the upregulation of the two lncRNA, MALAT1 and CASC2, in HCC tissue [19, 34–36]. In addition, MALAT1 was assessed in HCC patients’ serum [31] as well as among HCC patients with HCV infection [37]. CASC2 was also estimated in the serum of HCC patients on top of HCV by Refai and co-workers [38]. The current work was conducted to compare and evaluate the diagnostic efficacy of both MALAT1 and CASC2 biomarkers for HCC.

MALAT1 level in the serum of the control (healthy volunteer) represents the basal level of expression. The MALAT1 expression level was up-regulated in both of HCV patients’ group and the HCC/HCV patient group with the MALAT1 expression level higher in the HCV group than in the HCC/HCV group. A statistically significant difference was affirmed between the three groups. This comes in accordance with the previous findings of Konishi and his group as well as Toraih and coworkers [31, 37]. Also, this proposes MALAT1 as a promising marker for HCC diagnosis in HCV patients [31, 37]. This significant variation in MALAT1 expression level between HCC/HCV and HCV could be attributed to dissimilarities in fibrosis degree among the two groups [37]. MALAT1 is a key regulator in HCC and is closely related to tumor metastasis and recurrence. Studies have shown that MALAT1 can promote angiogenesis, thereby accelerating the development and metastasis of HCC [39, 40]. MALAT1 overexpression reduces the expression of miRNA-204, which leads to increased expression of SIRT1 and promotes the invasion and metastasis of HCC [39]. High expression of MALAT1 is also an independent predictor of HCC recurrence after liver transplantation [39, 40].

The CASC2 level in the serum of the control (healthy volunteer) was considered the basal level of expression. The CASC2 expression level was elevated in our HCV patient group and markedly decreased in the HCC/HCV patient group. In addition, a statistically significant difference between the three groups was matched with the previous report of Refai and his colleagues [38]. CASC2 is suggested as a marker for HCC diagnosis in HCV patients [38].

CASC2 acts as a tumor suppressor in HCC. Overexpression of CASC2 can promote apoptosis of HCC cells and inhibit cell proliferation, migration, and invasion [40, 41]. CASC2 can inhibit epithelial-mesenchymal transition (EMT) of HCC cells by sponging miR-367 and upregulating the expression of FBXW7 [41]. Additionally, CASC2 can improve the sensitivity of HCC cells to cisplatin by downregulating miR-222 [41].

Regarding the deregulation of the MALAT1 and CASC2 expression levels in our HCC\HCV group. We noticed that about half of the HCC patients had marked MALAT1 up-regulation. On the other hand, CASC2 downregulation was found in all of our HCC patients. Viral load in the HCV patient group showed wide variability with no significant correlation with a serum fold change of MALAT1 and CASC2 noticed.

ROC analysis was performed to determine the diagnostic efficacy and the cut‐off value for the LncRNAs MALAT1 and CASC2 and the routinely used biomarker AFP and their performance characteristics. The analysis clarified that CASC2 is better than MALAT1 in the prediction of the HCC among HCV patients, but we need to stress on CASC2 cut-off value that performs best accuracy and sensitivity is (0.995), which is near to the control (1.03**±**0.02) which are a healthy individual with no pre-hepatic condition but in case of our patient who had HCV infection (14.9±3.6) with the major part of them showed significant up-regulation will be markedly discriminating. The data also revealed that CASC2 has higher sensitivity and accuracy for HCC diagnosis than AFP. Accordingly, our study findings recommend CASC2 as a more promising diagnostic biomarker for HCC/HCV rather than MALAT1 or AFP.

Eventually, the limitation of our study is the small number of participants, so larger groups to prove different ethnicities are needed for comparisons and more exploration.

## Conclusion

According to our study results, the LncRNA CASC2 is a promising biomarker and is considered better and could help in HCC diagnosis on top of HCV than MALAT1 and AFP.

## Supporting information

S1 Graphical abstract (TIF)
