# Supplementary figures and images for: The clinical significance of long non-coding RNAs MALAT1 and CASC2 in the diagnosis of HCV-related hepatocellular carcinoma
Source: PLoS One. 2024 May 13;19(5):e0303314. doi: 10.1371/journal.pone.0303314 (PMC11090319; doi:10.1371/journal.pone.0303314)

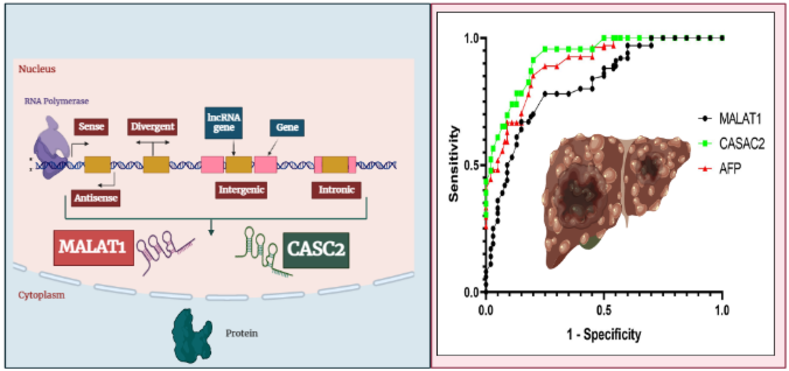

Supplement: S1 Graphical abstract — (TIF) [file pone.0303314.s001.tif]
